# A case of cervico-mediastinal paraganglioma mimicking an ectopic goiter

**DOI:** 10.1016/j.ijscr.2021.106357

**Published:** 2021-08-27

**Authors:** Daniel Bianchi, Adriana Scamporlino, Matteo Costantini, Giorgio Cavallesco, Uliano Morandi, Alessandro Stefani

**Affiliations:** a Division of Thoracic Surgery, University of Modena and Reggio Emilia, Modena, Italy; b Clinical and Experimental Medicine PhD Program, University of Modena and Reggio Emilia, Modena, Italy; c Department of Pathology, University Hospital of Modena, Modena, Italy; d Division of Thoracic Surgery, University of Ferrara, Ferrara, Italy

**Keywords:** Mediastinal paraganglioma, Ectopic goiter, Intrathoracic goiter, Surgery

## Abstract

**Introduction and importance:**

Mediastinal paragangliomas are rare neuroendocrine tumors that originate from extra-adrenal paraganglia, occasionally secreting catecholamines. Nonfunctional mediastinal paragangliomas present nonspecific clinical and radiological features and represent a diagnostic challenge.

**Case presentation:**

A 53-year old woman presented with cough and dyspnea increasing over time. CT-scan and ultrasonography showed a large vascularized cervico-mediastinal mass, consistent with an intrathoracic ectopic goiter. Preoperative angiography showed a blood supply from neck vessels. The lesion was completely removed through a cervical approach. The diagnosis of paraganglioma was a histological surprise. The patient is alive without recurrence 30 months after surgery.

**Clinical discussion:**

When preoperatively diagnosed, the treatment of choice of a mediastinal paraganglioma is surgical excision. However, a preoperative diagnosis of mediastinal paraganglioma is difficult to obtain, especially in cases of nonfunctional lesions. Distinction between an intrathoracic goiter and a nonfunctional paraganglioma can be extremely difficult and, given the rarity of the latter, an ectopic goiter is suspected in first instance. CT-scan and ultrasonography are of little use in the differential diagnosis. However, scintigraphy with ^123^I-metaiodobenzylguanidine can be an useful diagnostic tool when a paraganglioma is suspected. In case of vascularized cervico-mediastinal mass, such as paragangliomas or intrathoracic goiter, preoperative angiography should be performed to study the blood supply and orient the surgical approach.

**Conclusion:**

Although uncommon, paragangliomas should be considered in the differential diagnosis of mediastinal masses, especially when an ectopic goiter is suspected.

## Highlights

• Clinical diagnosis of mediastinal nonfunctioning paragangliomas is challenging.
• Mediastinal paragangliomas can mimic intrathoracic goiters.
• If a clinical suspicion of paraganglioma is raised, ^123^I-MIBG can be useful.
• In case of a vascularized cervico-mediastinal mass, an angiography is recommended.
• Angiography can detect the blood supply and guide the surgical approach.

## 1 Introduction

Paragangliomas are rare neuroendocrine tumors, originating from extra-adrenal paraganglia [1]. They are distinguished in functional and nonfunctional, depending on the hypersecretion of catecholamines. Mediastinal paragangliomas arise from mediastinal paraganglia and are extremely rare. They are usually located in the posterior mediastinum or in the aortopulmonary region and along the great vessels. Production of catecholamines is common in about half of the paragangliomas and the presence of the catecholamine syndrome may orient the diagnosis. In the literature, only 50 cases of functional mediastinal paragangliomas have been described [2]. The treatment of choice of mediastinal paragangliomas is surgical excision, usually through a thoracic approach [1]. In case of nonfunctional lesion, a preoperative diagnosis is difficult to achieve, since symptoms and radiological features are usually nonspecific and needle biopsy is often not feasible or not diagnostic [1].

Intrathoracic goiters are defined as multinodular goiters partially or totally located in the mediastinum. They are relatively common, accounting for 3–15% of all operated goiters and 6% of all mediastinal masses [3], [4]. Conversely, ectopic goiters consist of ectopic thyroid tissue anatomically separated from the thyroid gland, with an independent development [5] and represent only 1.7% of all intrathoracic goiters [3]. Ectopic goiters can be found in the anterior or in the visceral compartment of the mediastinum. The most common symptoms result from the compression of the adjacent structures and a thoracic approach is usually indicated for their removal [3].

We present a case of cervico-mediastinal nonfunctional paraganglioma mimicking an ectopic goiter. It was removed through a cervical approach, after a preoperative arteriographic study.

This work has been performed in line with SCARE 2020 criteria [6].

## 2 Case presentation

**Fig. 1:**
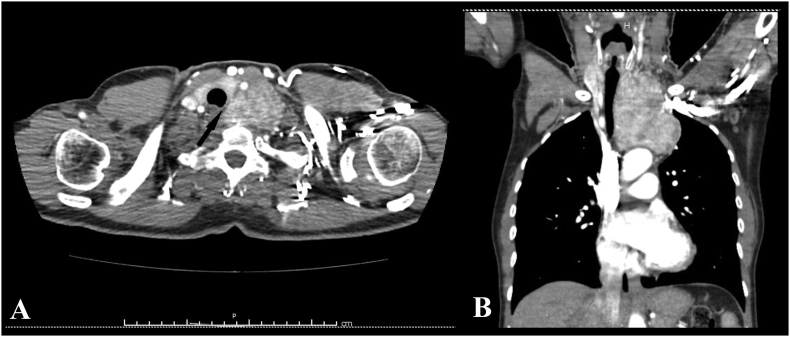
A, B. CT-scan showing a 90 × 80 × 50 mm mass, extending from the cervico-thoracic junction to the aortic arch. The lesion appears as highly vascularized and heterogeneous, contiguous but clearly separated from the cervical thyroid gland (arrow), which shows no abnormalities. Trachea, esophagus and left carotid and subclavian arteries are compressed and displaced.

A 53-year-old woman came to our observation for persistent cough and dyspnea, increasing over the last 12 months. On physical examination, swelling and venous distention in the left side of the neck were noted, together with dilated vein collaterals in the upper left hemithorax. Chest x-ray detected a mass in the upper anterior mediastinum, causing narrowing and right-side deviation of the trachea. Blood tests were normal, including thyroid function tests. The patient medical history was unremarkable. Contrast-enhanced CT-scan of the neck and thorax showed a mass in contiguity with the left thyroid lobe and extending in the upper mediastinum, consistent with an intrathoracic ectopic goiter (Fig. 1A, B). An ultrasound-guided needle biopsy of the mass was performed through the neck, but histological examination found red blood cells. The echographic appearance of the mass was also consistent with a goiter.

**Fig. 2:**
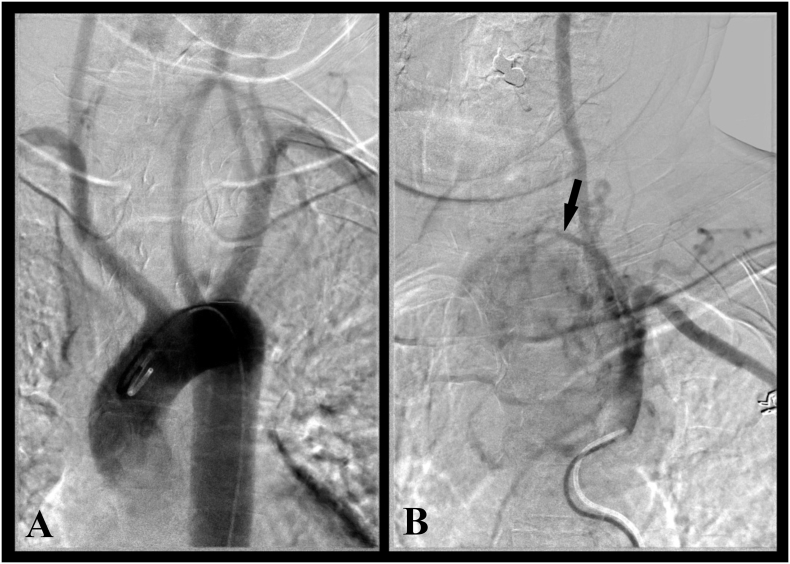
A, B. Arteriography showing a feeding vessel for the mass, originating from the inferior thyroid artery (arrow) and no blood supply from the mediastinum.

Surgical resection was indicated. Prior to surgery, arteriography was performed which showed a predominant blood supply from the neck and no significant feeding arteries coming from the mediastinum (Fig. 2A, B). Therefore, a cervical approach was preferred instead of a thoracic approach. UM and AS performed the procedure. A low transverse collar incision was performed. The mass appeared to be encapsulated, adherent to the surrounding structures but without signs of tissue infiltration. The thyroid gland was normal and the lesion appeared to be separated from the left thyroid lobe. The superior pole of the mass was clearly visible but the rest of the lesion extended deeply into the upper mediastinum. After ligation of the feeding vessels in the neck, gentle blunt dissection by finger and pledget was accomplished within the capsule, to achieve an adequate mobilization of the mass. Finally, the mass was delivered from the mediastinum and completely removed through the cervical incision. No significant bleeding occurred from the mediastinum. The left recurrent nerve was identified and preserved throughout the procedure. Frozen section examination found a mesenchymal tumor. The thyroid gland was not removed.

**Fig. 3:**
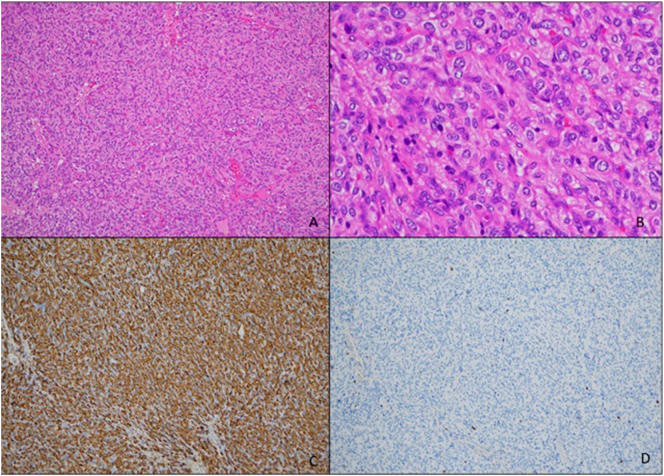
Microphotographs showing a neoplasm consisting of epithelioid cells with characteristic nested architecture, arranged in trabecolae and cords pattern [hematoxylin-eosin, 10× (A) and 40× (B)] diffuse positivity for synaptophysin (C) and low Ki67 proliferation index (D).

The post-operative course was uneventful and the patient was discharged on the fourth postoperative day. Histological examination showed a paraganglioma with a low mitotic index <5% (Fig. 3). The resection was complete. The patient is alive without recurrence 30 months after surgery.

## 3 Discussion

Mediastinal paragangliomas are rare entities, usually located in the posterior mediastinum or in the aortopulmonary window and along the great vessels. The presence of the catecholamine syndrome may orient the diagnosis of a mediastinal mass toward a paraganglioma. Conversely, nonfunctional mediastinal paragangliomas represent a diagnostic challenge. They are extremely uncommon and symptoms are only related to mass effect [7]. At CT-scan, paraganglioma presents as a well-defined mass with high contrast enhancement and with areas of lower attenuation representing necrosis, but these features are quite nonspecific among mediastinal masses. Needle biopsy can be hazardous, due to the proximity of the tumor to the great vessels and to its vascularization, and has a low diagnostic accuracy. A complete pathological examination is usually required to achieve a diagnosis [7], [8].

When preoperatively diagnosed, the treatment of choice of a mediastinal paraganglioma is surgical excision [8]. However, the nonfunctional forms are usually diagnosed only at pathological examination after removal [1]. In the reported case symptoms were nonspecific, the location of the mass and CT-scan and ultrasound features oriented the diagnosis toward an ectopic goiter. The needle biopsy was not representative of the lesion. However, surgical excision was clearly indicated, to relief compressive symptoms. Intraoperatively, the macroscopic appearance of the tumor was also consistent with a goiter and the pathological diagnosis of paraganglioma was definitely unexpected.

An ectopic goiter represents undoubtedly the favored diagnosis when approaching a mass presenting as in our patient. It is difficult to take into account a paraganglioma in the differential diagnosis, especially in the absence of a catecholamine syndrome. However, when a paraganglioma is suspected, scintigraphy with ^123^I-metaiodobenzylguanidine (^123^I-MIBG) could be used [1]. This technique is highly sensitive and specific for pheochromocytomas and paragangliomas of all types and locations, including nonfunctional forms. ^123^I-MIBG is preferable to somatostatin receptor scintigraphy and to positron emission tomography, which can be positive also in case of goiter.

When surgery for a cervico-mediastinal mass is planned, the choice of the approach, either from the neck or the thorax, is crucial. Especially in case of vascularized lesions, the approach depends primarily on the origin of the feeding vessels. The preferred approach for an ectopic goiter is through the thorax (thoracotomy or sternotomy), because the blood supply usually comes from mediastinal vessels and the removal through a cervical incision may be dangerous. Only a few cases of ectopic goiters which were removed from the neck have been reported [3]. The blood supply of mediastinal paragangliomas is generally from the mediastinum as well, therefore a thoracic approach provides an adequate access. To the best of our knowledge, only another case of mediastinal paraganglioma removed through a cervical approach has been reported [9]. When CT-scan does not clearly detect the origin of the blood supply, an arteriography should be performed preoperatively to guide the choice of the surgical approach. In our case, arteriography showed that the predominant blood supply came from the inferior thyroid artery. This finding led us to choose a cervical access, even if we thought we were approaching an ectopic mediastinal goiter.

## 4 Conclusions

Nonfunctional mediastinal paragangliomas are very uncommon and represent a diagnostic challenge. This case report contributes for some aspects to the knowledge of this entity. When presenting as cervico-mediastinal masses, paragangliomas can be easily misdiagnosed as intrathoracic or ectopic goiters. If a clinical suspicion of paraganglioma is raised, ^123^I-MIBG can be useful for the differential diagnosis. In case of a vascularized cervico-mediastinal mass, a preoperative angiographic study is recommended to detect its blood supply. The surgical approach of such lesions should be guided by the origin of the feeding vessels, regardless of the diagnostic suspicion. When completely removed, paragangliomas do not recur.

## Consent

Written informed consent was obtained from the patient for publication of this case report and accompanying images. A copy of the written consent is available for review by the Editor-in-Chief of this journal on request.

## Provenance

Not commissioned.

## Peer review

Externally, peer-reviewed.

## Ethical approval

Ethical board approval is not required for a single case report in our Center.

## Funding

This research did not receive any specific grant from funding agencies in the public, commercial, or not-for-profit sectors.

## Guarantor

Alessandro Stefani is the guarantor of this study.

## Research registration number

Not applicable.

## CRediT authorship contribution statement

Daniel Bianchi and Matteo Costantini collected data.

Alessandro Stefani and Daniel Bianchi wrote the manuscript.

Adriana Scamporlino, Giorgio Cavallesco and Uliano Morandi revised and approved the manuscript.

## Declaration of competing interest

None.
